# Surveillance of Avian H7N9 Virus in Various Environments of Zhejiang Province, China before and after Live Poultry Markets Were Closed in 2013–2014

**DOI:** 10.1371/journal.pone.0135718

**Published:** 2015-08-26

**Authors:** Xiaoxiao Wang, Shelan Liu, Haiyan Mao, Zhao Yu, Enfu Chen, Chengliang Chai

**Affiliations:** 1 Zhejiang Provincial Centre for Disease Control and Prevention, Hangzhou, People’s Republic of China; 2 Field Epidemiology Training Program of Zhejiang Province, Hangzhou, People’s Republic of China; Deutsches Primatenzentrum GmbH—Leibniz-Institut fur Primatenforschung, GERMANY

## Abstract

**Background:**

To date, there have been a total of 637 laboratory-confirmed cases of human infection with avian influenza A (H7N9) virus across mainland China, with 28% (179/637) of these reported in Zhejiang Province. Surveillance of avian H7N9 virus was conducted to investigate environmental contamination during H7N9 outbreaks. We sought to evaluate the prevalence of H7N9 in the environment, and the effects of poultry market closures on the incidence of human H7N9 cases.

**Methods:**

We collected 6740 environmental samples from 751 sampling sites across 11 cities of Zhejiang Province (China) between January 2013 and March 2014. The presence of H7N9 was determined by reverse transcription polymerase chain reaction, with prevalence compared between sites and over time. The relationship between environmental contamination and human cases of H7N9 infection were analyzed using Spearman’s ranked correlation coefficient.

**Results:**

Of the 6740 samples, 10.09% (680/6740) were H7N9-positive. The virus was found to circulate seasonally, and peaked during the spring and winter of 2013–2014. The prevalence of the virus decreased from the north to the southeast of the province, coinciding with the geographical distribution of human H7N9 cases. Compared with other sampling sites, live poultry markets (LPMs) had the highest prevalence of H7N9 virus at 13.94% (667/4784). Of the various sample types analyzed, virus prevalence was highest for chopping board swabs at 15.49% (110/710). The prevalence of the virus in the environment positively correlated with the incidence of human H7N9 cases (r^2^ = 0.498; *P* < 0.01). Cities with a higher incidence of human H7N9 cases also had a higher prevalence of H7N9 among samples and at sampling sites. Following the closure of LPMs at the end of January 2014, the prevalence of H7N9 decreased from 19.18% (487/2539) to 6.92% (79/1141). This corresponded with a decrease in the number of human H7N9 cases reported.

**Conclusions:**

The prevalence of H7N9 virus in environmental samples oscillated seasonally, regardless of whether LPMs were open. The presence of H7N9 in environmental samples positively correlated with the number of human H7N9 cases, indicating that eradication of the virus from the environment is essential in reducing the numbers of H7N9 cases and halting the spread of the virus.

## Introduction

Avian influenza, caused by the reassorted subtype avian influenza A (H7N9) virus, was first confirmed in Shanghai (China) on 31 March 2013. Between 2013 and 2015, this virus had spread to 17 provinces of mainland China, with 637 cases reported up until 29 April 2015. A large proportion of cases were located in the east and south of China along the coast. Zhejiang Province, which is near Shanghai municipality, had the highest prevalence of H7N9 among all provinces. A total of 179 cases were identified between April 2013 and April 2015[1–3]. Of the 11 cities in Zhejiang Province that were sampled, H7N9 human cases were reported in 10[4–7].

It has been reported that AIVs can survive for a few days in feces, or for several months in water[8]. AIV existed in environmental samples and in infected poultry during influenza A (H5N1) outbreaks[9, 10]. Epidemiological data suggested that avian influenza H7N9 infections were associated with exposure to live poultry markets (LPMs). Incidences of human H7N9 infection have decreased substantially since the closure of LPMs, further suggesting that the primary risk factor for H7N9 infection is exposure to live birds. It has been postulated that recombination and dissemination of avian influenza viruses (AIVs) occurs in LPMs[11–16].The surveillance of LPMs has been conducted in several countries, including China, to detect circulating influenza viruses. Active environmental surveillance in Zhejiang Province has been carried out since 2011. Emergency surveillance took place in 2013–2014, as part of the control measures instituted during a number of avian influenza A (H7N9) outbreaks. We focused on the continuous and systematic surveillance of AIV H7N9 in the environment. Multiple sampling sites throughout Zhejiang Province, besides LPMs, were examined. The majority of LPMs were closed at the end of January 2014, with some LPMs in southern counties open until mid-February 2014. Only LPMs in urban areas were required to close, with those in rural areas allowed to remain open. The LPMs and their surrounding environs in urban areas were thoroughly cleaned after their closure, and not sampled again. We investigated the potential relationship between the incidence of human H7N9 cases, closure of LPMs, and the presence of H7N9 in various environments. The findings from our study might provide us with better knowledge regarding levels of environmental contamination with H7N9, allowing us to develop targeted control interventions and improve environmental surveillance strategies.

## Methods

### Ethics Statement

Ethical approval for patients’ data collection and environment sampling was not required in this study: a) no specific permissions were required for these locations; b) these locations are not privately-owned or protected; c) activities did not involve endangered or protected species. Environmental surveillance was conducted as part of control and management for national notifiable diseases. And it was determined by the National Health and Family Planning Commission that the collection of data from H7N9 cases was part of investigations of an emerging outbreak and was exempt from institutional review board assessment[17]. The use of data in our study was approved by medical ethical committee. Epidemiological data of patients were collected by local center for disease control and prevention (CDC) staff and reported to Zhejiang Provincial CDC and China CDC through Chinese Information Systems for Disease Control and Prevention. Patient information was not anonymized in this system, but was anonymized before analysis.

### Environmental Surveillance

#### (1) Routine and emergency surveillance

For the routine surveillance program, samples were collected from 11 cities and their surrounding environs. Three or four cities were randomly selected each month to collect more than 40 environmental samples. Emergency environmental surveillance programs were implemented in areas with confirmed cases of AIV H7N9 infection in April 2013. Specifically, 20–30 samples were collected from each county in the cities of Hangzhou, Huzhou and Jiaxing. For a further eight cities, one county was randomly selected for sampling. In 2014, more than 30 samples were collected from each city every month from the various sampling sites across Zhejiang Province. We obtained 6740 samples from January 2013 through to March 2014.

#### (2) Sources and types of environmental sample

The sampling sites we investigated included LPMs, poultry breeding farms, backyard poultry pens, poultry processing factories, and wild bird habitats. Six different sample types were collected: cage surface swabs; chopping board surface swabs; feces samples; water for cleaning; drinking water; and surface swabs of equipment. Each city was not required to collect all the six different sample types at each sampling sites each month, but was recommended to collect as many as possible.

#### (3) Sample collection, transportation, storage and laboratory testing

As outlined in previous studies, environmental samples were aliquoted into three cryovials after collection and transported to a World Health Organization Influenza Network Laboratory within 48 h at 4°C. Cryovials were stored at −70°C until required[10]. Qiagen RNeasy Mini Kits (Hilden, Germany) were used to extract RNA from samples according to the manufacturer’s instructions. Quantitative real-time reverse transcription polymerase chain reaction (qPCR) assays were used to detect influenza A H7N9, employing primers FluA-Forward (5′-GAC CRA TCC TGT CAC CTC TGA C-3′) and FluA-Reverse (5′-GGG CAT TYT GGA CAA AKC GTC TAC G-3′) and a specific probe, FluA-Probe (5′-TGC AGT CCT CGC TCA CTG GGC ACG-3′)[2, 3]. To confirm our results, all samples were also tested, using PCR methods, by the Zhejiang Provincial CDC.

### H7N9 case surveillance and investigation

Patients’ data including dates regarding the onset of illness and their exposure history to poultry or wild birds were collected from Chinese Information System for Disease Control and Prevention. For those that had visited LPMs or been in contact with backyard poultry in the 2 weeks prior to the onset of illness, those sites were considered “sites epidemiologically relating to incidences”. The others were defined as “sites not visited by H7N9 cases during the incubation period”.

To determine the association between environmental H7N9 virus contamination and case distribution, we sorted 11 cities in Zhejiang Province according to the number of cases identified from January 2013 to March 2014.Cities with more than ten H7N9 cases included Hangzhou, Ningbo and Shaoxing. Between one and ten cases were identified in Huzhou, Jiaxing, Jinhua, Lishui, Taizhou, and Wenzhou, while no confirmed cases were apparent in Quzhou and Zhoushan.

### Statistical Analysis

Maps indicating H7N9-positive sites and cases were generated using Epi-info 3.5 (US CDC, Atlanta, GA, USA). The chi-squared test was used to compare H7N9 virus prevalence among sample types, epidemiological seasons, and cities. Spearman’s rank correlation coefficient was used to analyze prevalence of the virus in environment samples against infection cases. All statistical tests were conducted with SPSS version 21.0 (SPSS Inc, Chicago, IL, USA) For two-sided tests, a P-value less than 0.05 was considered statistically significant.

## Results

### Seasonal distribution of H7N9 prevalence in environmental samples

**Fig 1:**
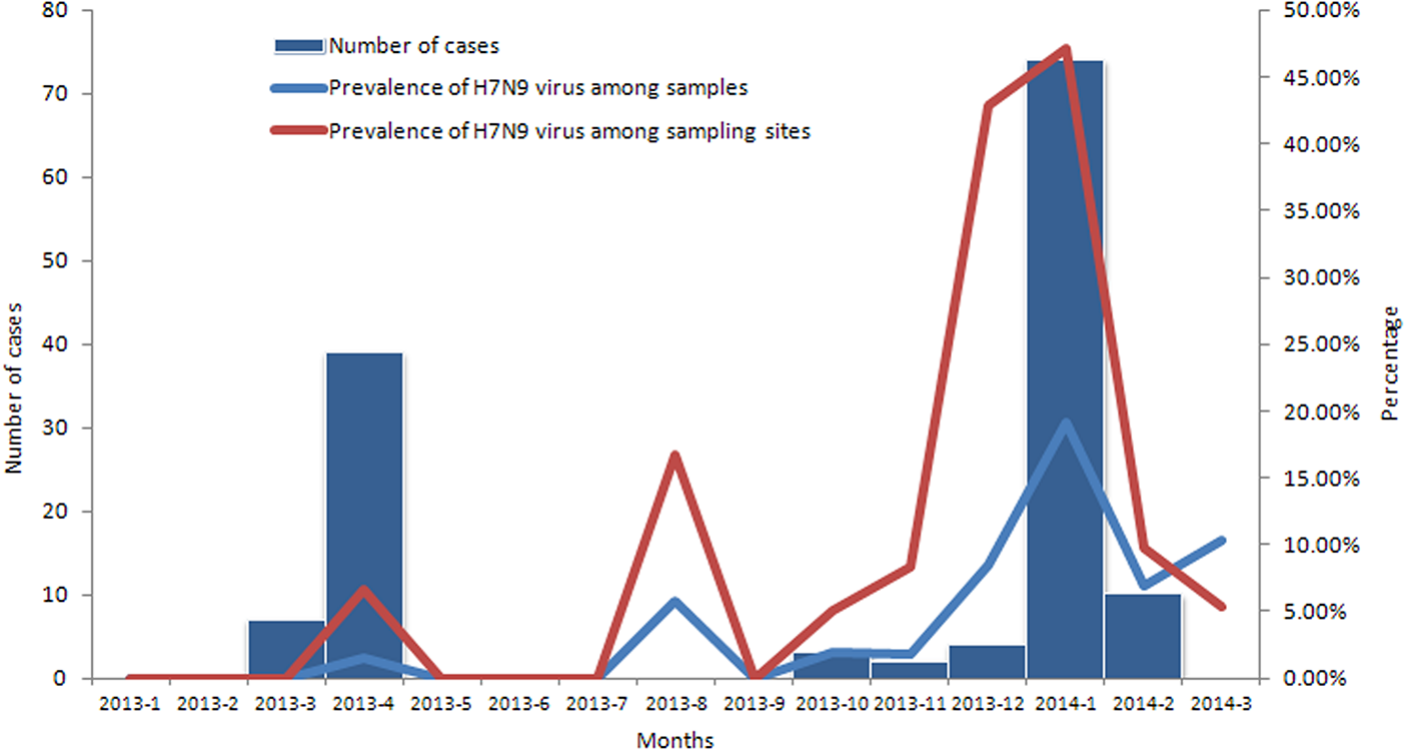
Numbers of cases according to onset of illness and prevalence of H7N9 among samples and at sampling sites during January 2013 to March 2014.

In 2013, 2458 samples were collected from 404 sampling sites, with 2.12% (52/2458) of samples and 10.89% (44/404) of sites positive for H7N9 virus. During the H7N9 outbreak in April 2013, 1.46% (15/1029) of specimens from nine sampling sites in eight cities tested positive for H7N9. In August, October, and November of 2013, very few samples were H7N9-positive, however in December 2013, the prevalence of H7N9 increased to 8.50% (21/247). In January 2014, the prevalence of H7N9 virus-positive samples peaked at 19.18% (487/2539) when AIV H7N9 re-emerged in Zhejiang Province, declining to 6.92% (79/1141) in February, and then increasing to 10.30% (62/602) in March 2014 (Fig 1; S1 Table).

### Spatial distribution of H7N9 virus in Zhejiang Province

**Fig 2:**
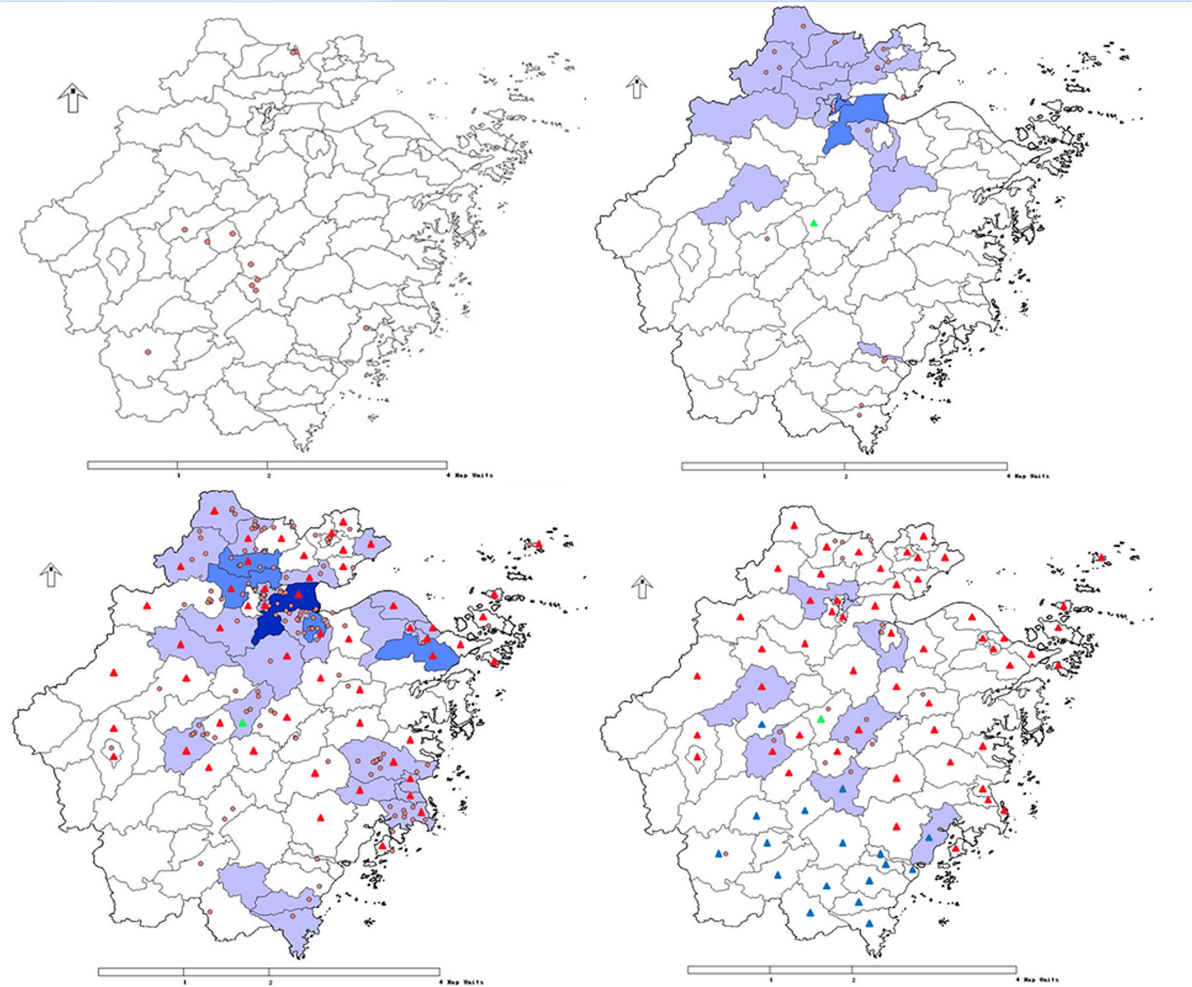
Geographical distribution of H7N9 positive samples and confirmed cases in 2013(upper left), January(upper right), February(lower left) and March(lower right), 2014 Red circle: The sampling site with positive H7N9 virus Red triangle: Counties with live poultry markets closed at the end of January, 2014 Blue triangle: Counties with live poultry markets closed in February, 2014 Green triangle: Counties with live poultry markets closed in July, 2013 Light blue:Counties with 1~5 cases confirmed Blue:Counties with 5~10 cases confirmed Dark blue:Counties with >10 cases confirmed.

In 2013, 13 districts in seven cities were H7N9-positive. In the 13 districts, there were six reported cases of H7N9 infection. The H7N9-positive sites and cases exhibited similar spatial distribution, and were mainly located in the north of Zhejiang Province. In January 2014, 40 districts in 11 cities of Zhejiang Province had sites that were contaminated with H7N9. These areas were mainly located in the north, central, and southeast regions of the province. In February and March of 2014, more than 10 districts in central Zhejiang Province tested positive for H7N9. From 2013 to 2014, the areas contaminated by H7N9 expanded from the north of Zhejiang Province to central and southeastern regions. The distribution of H7N9-contaminated sites was consistent with the location of H7N9 infection cases (Fig 2).

### H7N9 prevalence among sample types and sampling sites

The prevalence of H7N9 in LPMs was the highest of all sampling sites surveyed, at 3.76% (48/1277) to 21.07% (59/280) for 2013–2014 (Table 1). Poultry processing factories were also highly contaminated, with a prevalence of 16.67% (1/6) from February 2014. Samples from backyard poultry pens and wild bird habitats tested negative for the presence of AIV H7N9. The prevalence of the virus in samples from poultry farms was 0.55% (4/733) and 4.76% (5/105) for 2013 and January 2014, respectively. Prior to their closure, H7N9 virus was predominantly found in urban LPMs. After their closure, H7N9 was then detected in LPMs of rural areas and towns, and in poultry processing factories, although the prevalence of the virus in poultry processing factories was lower than that in LPMs (Table 1).

**Table 1:** Prevalence of H7N9 virus among samples from different sampling sites between January 2013 and March 2014.

Period	Poultry markets	Poultry farms	Backyard poultry pens	Poultry processing factories	Wild bird habitats
January-December 2013	3.76%(48/1277)	0.55%(4/733)	0%(0/335)	0%(0/25)	0%(0/98)
January 2014	20.81%(482/2316)	4.76%(5/105)	0%(0/103)	0%(0/6)	0%(0/9)
February 2014	8.47%(78/921)	0%(0/117)	0%(0/97)	16.67%(1/6)	0%(0/0)
March 2014	21.07%(59/280)	0%(0/156)	0%(0/114)	16.67%(3/18)	0%(0/5)
Total	13.94%(667/4784)	0.81%(9/1111)	0%(0/649)	7.27%(4/55)	0%(0/112)

The prevalence of H7N9 virus in the various types of samples analyzed is summarized in Table 2. The prevalence of the virus in 2013–2014 was 4.43% (7/158) to 23.53% (80/340) for chopping board surface swabs, while the lowest levels of prevalence were evident for fecal samples (7.07%, 184/2604). For 2013 and 2014, the differences in prevalence between sample types were statistically significant (*P <* 0.01). Between January 2013 and January 2014, when LPMs were open, the prevalence of H7N9 was highest for chopping board surfaces and cage surfaces. Once LPMs had closed, H7N9 prevalence was highest in drinking water samples in February 2014, and in water used to clean poultry in March 2014.

**Table 2:** Prevalence of H7N9 virus among sample types obtained between January 2013 and March 2014.

Period	Cage surface swabs	Chopping board surface swabs	Feces	Water for cleaning	Drinking water	Swabs of other surfaces	χ^2^	*P*-value
2013	1.13%(6/530)	4.43%(7/158)	2.26%(27/1193)	2.91%(5/172)	1.04%(3/289)	5.17%(6/116)	13.48	<0.05
January 2014	23.05%(151/655)	23.53%(80/340)	14.65%(116/792)	20.47%(61/298)	16.39%(39/238)	18.52%(40/216)	22.57	**<0.01**
February 2014	4.38%(13/297)	9.92%(13/131)	3.88%(14/361)	5.71%(8/140)	18.32%(24/131)	8.64%(7/81)	36.37	**<0.01**
March 2014	3.81%(4/105)	12.35%(10/81)	10.47%(27/258)	21.88%(14/64)	9.23%(6/65)	3.45%(1/29)	16.00	**<0.01**
Total	10.96%(174/1587)	15.49%(110/710)	7.07%(184/2604)	13.06%(88/674)	9.96%(72/723)	11.76%(52/442)	58.35	**<0.01**

### Prevalence of H7N9 in the environment correlated with the distribution of human H7N9 cases and closure of LPMs

During the H7N9 avian influenza outbreaks in March and April of 2013, virus-positive samples were identified at sites where human cases of H7N9 infection were confirmed. Virus prevalence was significantly higher at these sites than those where no human H7N9 cases were confirmed (*P* < 0.01). Between December 2013 and February 2014, when avian influenza H7N9 re-emerged, virus prevalence at sites where human H7N9 cases were confirmed was comparable with that at sites where no human H7N9 cases were reported. A comparison of the outbreaks in 2013 and 2014 revealed similar levels of H7N9 prevalence at sites where human cases of H7N9 infection were reported. However, for sites that were not associated with human cases of H7N9 infection, a greater proportion of samples were contaminated with virus in 2014 than in 2013(*P* < 0.01; Table 3).

**Table 3:** A comparison of H7N9 virus prevalence in sites associated with human cases of H7N9 infection.

Period	Sites epidemically related to the incidence of human H7N9 infection	Sites not epidemically related to the incidence of H7N9 infection	χ^2^	*P*-value
March 2013 to April 2013	12.60%(16/127)	2.16%(13/603)	29.987	**<0.01**
December 2013 to February 2014	19.62%(62/316)	17.20%(536/3116)	1.167	>0.05
χ^2^	3.079	90.89		
*P*-value	>0.05	**<0.01**		

The prevalence of H7N9 virus in the environment, and human H7N9 case distribution exhibited similar spatial trends. For 2014, cities with confirmed human cases of H7N9 virus infection had a higher prevalence of H7N9 than those without human cases (Table 4). In March 2014, samples from cities with greater than 10 confirmed human H7N9 cases were negative for the presence H7N9, as were samples from cities where no human cases of infection were reported. However, 12.84% (62/483) of samples and 9.38% (3/32) of sampling sites were H7N9-positive in cities with 1–10 confirmed cases of human infection. A correlation coefficient of 0.498 was calculated for prevalence of H7N9 in the environment and case numbers during H7N9 outbreaks in April 2013, October–November 2013, and January–February 2014 (*P <* 0.01).

**Table 4:** Prevalence of H7N9 rates among samples and sampling sites from cities in Zhejiang Province.

Period		Cities with >10 cases of human H7N9 infection	Cities with 1–10 cases of human H7N9 infection	Cities without cases of human H7N9 infection	χ^2^	*P*-value
January	Prevalence of H7N9 among samples	31.21%(221/708)	16.60%(260/1566)	2.26%(6/265)	121.721	**<0.01**
	Prevalence of H7N9 at sampling sites	58.65%(61/104)	36.19%(76/210)	9.68%(3/31)	27.852	**<0.01**
February	Prevalence of H7N9 among samples	1.54%(5/325)	9.99%(74/741)	0%(0/75)	6.805	**<0.01**
	Prevalence of H7N9 at sampling sites	6.35%(4/63)	13.58%(11/81)	0%(0/9)	0.355	>0.05
March	Prevalence of H7N9 among samples	0%(0/69)	12.84%(62/483)	0%(0/50)	-	-
	Prevalence of H7N9 at sampling sites	0%(0/11)	9.38%(3/32)	0%(0/13)	-	-
Total	Prevalence of H7N9 among samples	20.51%(226/1102)	14.19%(396/2790)	1.54%(6/390)	77.687	**<0.01**
	Prevalence of H7N9 at sampling sites	36.52%(65/178)	27.86%(90/323)	5.66%(3/53)	16.764	**<0.01**

Following the closure of most LPMs, the prevalence of H7N9 virus among samples decreased from 19.18% (487/2539) in January 2014 to 6.92% (79/1141) in February 2014, and from 47.10% (65/138) to 9.80% (15/153) for sampling sites. At the same time, the number of human H7N9 infection cases declined from seventy-four to ten. After LPMs in southern cities were also closed in mid-February 2014, the prevalence of H7N9 among samples in March 2014 increased to 10.30% (62/602), while that for sampling sites dropped to 5.36% (3/56), with no further confirmed cases of infection reported.

## Discussion

Experimental studies and field investigations have shown that AIV is frequently present on various surfaces of influenza-associated sites and their surrounding environments[9, 18]. Therefore, environments contaminated with AIV can act as potential sources of virus transmission from poultry to humans. In China, more than 80% of human H7N9 cases had reported being exposed to poultry before the onset of illness, with 68% of cases having LPM exposure[19–22]. In Zhejiang Province, 86.81% of human cases had been exposed to LPMs, or poultry in another setting, highlighting the effects of environmental contamination. In Shanghai, because of six human cases of H7N9 infection, 280 environmental samples and swabs from LPMs were collected from surrounding areas, with 7.14% (20/280) positive for H7N9[23]. Another survey conducted in the southern Chinese city of Guangzhou revealed that 44.4% (16/36) and 50.0% (3/6) of retail and wholesale LPMs, respectively were contaminated with AIV H7N9[24]. In the current study, we observed a higher prevalence of H7N9 virus in Zhejiang Province than that in Shanghai and Guangzhou; these findings could be crucial for risk assessment, and the prediction of future AIV outbreaks.

The prevalence of H7N9 virus in the environment increased in spring and winter, exhibiting typical seasonal distributions. This seasonal pattern was consistent with the incidence of human H7N9 infections in Zhejiang Province, and with incidences of seasonal influenza and H5N1 infection in humans. The reasons for this seasonality could be because the average temperatures in spring and winter were suitable for virus. Previous reports indicate that humidity, temperature, and other weather conditions affect the incidence of influenza and human H5N1[25–27]; therefore H7N9 could also be affected by these factors. In addition, there are higher levels of exposure to live poultry in spring and winter, especially during the Chinese New Year period, with live poultry favored in most areas of China.

We also found that prevalence of H7N9 among samples types was similar to that for H5N1[28–30]. First, the prevalence of H7N9 was significantly lower in fecal samples than in all other sample types in our study. This finding was consistent with influenza A (H5N1) surveillance outcomes from LPMs of Cambodia[28]. Second, H7N9 prevalence was very high for chopping board surface swabs. Slaughtering birds in markets was also a risk factor for avian influenza (H5N1) infection in Indonesia[29, 30]. This was significant with respect to our results, because chopping boards are used at the final stage of poultry slaughtering. Raw poultry that comes in contact with contaminated chopping boards poses a significant risk to consumers, allowing for the transmission of viruses. According to the prevalence of AIV (H5N1) in LPMs, bans on keeping live poultry in markets overnight, rest days, daily cage cleaning, and disinfecting LPMs were instigated to reduce the spread of avian influenza (H5N1)[31–33]. For H7N9, these measures might be also suitable.

In our study, positive samples from 2013 and January 2014 were predominantly from LPMs, while those from February and March 2014 were from both LPMs and poultry processing factories. LPMs and poultry processing factories were the most contaminated facilities in Zhejiang Province during the H7N9 outbreaks. Our findings were similar to those reported in Indonesia regarding AIV H5N1, where sale zones and slaughter zones were the areas that were most commonly contaminated[29, 30]. Our results showed that poultry farms were also H7N9-positive; however it was more efficient to monitor AIV H7N9 at LPMs and poultry processing factories. The high prevalence of H7N9 might be because of the mixture of different poultry types in LPMs and poultry processing factories, and/or because equipment such as cages, chopping boards, and tools for removing feathers are shared. Furthermore, these environments are generally contaminated with feathers and fecal matter, and subject to poor ventilation, which can help the H7N9 virus to survive. Given that the virus was present in sites other than LPMs, their closure might not be the only means of halting the spread of H7N9. Other means of controlling H7N9 spread should be investigated, such as routine cleaning and disinfection of equipment and environment in poultry farms, poultry processing factories.

Our findings support an association between environmental contamination with H7N9 and human cases of H7N9 infection. In spring 2013, samples from sites associated with human cases of H7N9 infection had a higher prevalence of virus than those from sites not associated with cases. However, between December 2013 and February 2014, viral prevalence was comparable between sites regardless of whether they were associated with human cases of infection. This particular lack of difference could be related to the widespread outbreak of avian influenza A (H7N9) across Zhejiang Province, with most LPMs and sampling sites contaminated with the virus.The prevalence of H7N9 among samples and sampling sites obtained from January to March 2014 clearly showed that areas with confirmed cases of human H7N9 infection were more commonly contaminated with virus than those without cases. Areas without reported cases of H7N9 infection (Quzhou and Zhoushan) were found to be contaminated with virus in January 2014, when the prevalence of AIV H7N9 peaked. However, virus prevalence among samples and sample sites was low, and had remained low since February. Interventions and control measures, such as the closure of LPMs after outbreaks in other cities might have prevented H7N9 from spreading to Quzhou and Zhoushan. Further research is required to understand the reason why no cases were reported in these two cities, and might help us respond to potential outbreaks in the future.

There were several limitations to our study. First, during the environmental surveillance in 2013, only three or four cities were randomly selected to collect samples each month, with the exception of April. Second, according to our emergency environmental surveillance programs, more samples were collected from cities in which human cases of H7N9 were identified than in those without, therefore the number of samples from each city was unbalanced. Third, virus isolation was not conducted to confirm our PCR results, which might result in some bias regarding the reported prevalence rates.

In conclusion, we demonstrated that the prevalence of H7N9 virus in the environment positively correlated with the occurrence of human H7N9 cases. Environmental contamination with H7N9 oscillated seasonally, peaking in spring and winter regardless of whether LPMs were open. LPMs and poultry processing factories were consistently contaminated with H7N9, suggesting that they should be monitored on a regular basis for the presence of virus. Prevalence of H7N9 among sample types was similar with that for H5N1, indicating measures for reducing H5N1 in the environment might also be effective against H7N9. After the closure of LPMs, measures conducted to limit the spread of AIV H7N9 in environments other than LPMs are essential for reducing the incidence of human H7N9 infections.

## Supporting Information

S1 Table Prevalence of H7N9 virus among samples and sampling sites between January 2013 and March 2014. (DOCX)

S2 Table Geographical coordinates of 11 cities for environmental surveillance in the study. (DOCX)
