# Supplementary material for: Surveillance of Avian H7N9 Virus in Various Environments of Zhejiang Province, China before and after Live Poultry Markets Were Closed in 2013–2014
Source: PLoS One. 2015 Aug 26;10(8):e0135718. doi: 10.1371/journal.pone.0135718 (PMC4550274; doi:10.1371/journal.pone.0135718)
Supplement: S1 Table — (DOCX) [file pone.0135718.s001.docx]

**S1 Table** Prevalence of H7N9 virus among samples and sampling sites between January 2013 and March 2014

| Year | Month | Prevalence of H7N9 virus among samples | Prevalence of H7N9 virus among sampling sites |
| --- | --- | --- | --- |
|  | January | 0%(0/58) | 0%(0/8) |
|  | February | 0%(0/32) | 0%(0/5) |
|  | March | 0%(0/102) | 0%(0/32) |
|  | April | 1.46%(15/1029) | 6.62%(9/136) |
|  | May | 0%(0/76) | 0%(0/11) |
|  | June | 0%(0/61) | 0%(0/15) |
| 2013 | July | 0%(0/59) | 0%(0/10) |
|  | August | 5.77%(3/52) | 16.67%(1/6) |
|  | September | 0%(0/60) | 0%(0/11) |
|  | October | 1.99%(6/302) | 5.08%(3/59) |
|  | November | 1.84%(7/380) | 8.33%(4/47) |
|  | December | 8.50%(21/247) | 42.86%(27/63) |
|  | Subtotal | 2.12%(52/2458) | 10.89%(44/404) |
|  | January | 19.18%(487/2539) | 47.10%(65/138) |
| 2014 | February | 6.92%(79/1141) | 9.80%(15/153) |
|  | March | 10.30%(62/602) | 5.36%(3/56) |
|  | Subtotal | 14.67%(628/4282) | 23.92%(83/347) |
| Total | | 10.09%(680/6740) | 16.91%(127/751) |
