# Supplementary material for: Surveillance of Avian H7N9 Virus in Various Environments of Zhejiang Province, China before and after Live Poultry Markets Were Closed in 2013–2014
Source: PLoS One. 2015 Aug 26;10(8):e0135718. doi: 10.1371/journal.pone.0135718 (PMC4550274; doi:10.1371/journal.pone.0135718)
Supplement: S2 Table — (DOCX) [file pone.0135718.s002.docx]

S2 Table Geographical coordinates of 11 cities for environmental surveillance in the study

| Cities | Geographical coordinates |
| --- | --- |
| Hangzhou | 30.16°N,120.10°E |
| Ningbo | 29.52°N,121.33°E |
| Jiaxing | 30.46°N,120.45°E |
| Huzhou | 30.52°N,120.06°E |
| Shaoxing | 30.00 °N,120.34°E |
| Wenzhou | 28.01 °N,120.39°E |
| Taizhou | 28.41 °N,121.27°E |
| Jinhua | 29.07 °N,119.39°E |
| Quzhou | 28.58 °N,118.52°E |
| Lishui | 28.27 °N,119.54°E |
| Zhoushan | 30.01 °N,122.06°E |
